# Supplementary material for: From Data to Diagnosis: How Machine Learning Is Changing Heart Health Monitoring
Source: Int J Environ Res Public Health. 2023 Mar 5;20(5):4605. doi: 10.3390/ijerph20054605 (PMC10002005; doi:10.3390/ijerph20054605)

Methods: "The PubMed, Scopus and Goggle Scholar search engines and the Espacenet and Patentscope patent databases were used in the analysis, using the following keywords: combination of "deep learning", "machine learning" "ML", "artificial intelligence". "AI" with "heart sensor", "electrocardiography", "ECG", photoplethysmography", "PPG". Article exclusion factors were manuscripts written in a language other than English, lack of ECG or PPG data or machine learning methods involved in the study, articles published before 2018 (with a few exceptions of articles essential to the development of the techniques described). At the same time, to ensure originality and accuracy, the search focused on research papers, while excluding review articles. As a result, items such as papers with titles containing words such as 'review', 'study', 'meta-analysis' or 'overview' were not included.

Results: "1686 articles and patents published were identified. The sources were divided into two distinct groups: ECG and PGG. The full analysis according to the PRISMA 2020 methodology is presented in the Supplementary material. The paper presents the most important developments in the use of machine learning methods as a support for accurate ECG and PPG-based heart rate analysis. It also outlines the most important research directions that should be pursued in the near future, including ensuring the accuracy of measurement and the real-time interpretation of the results, as well as validating the data in clinical trials."

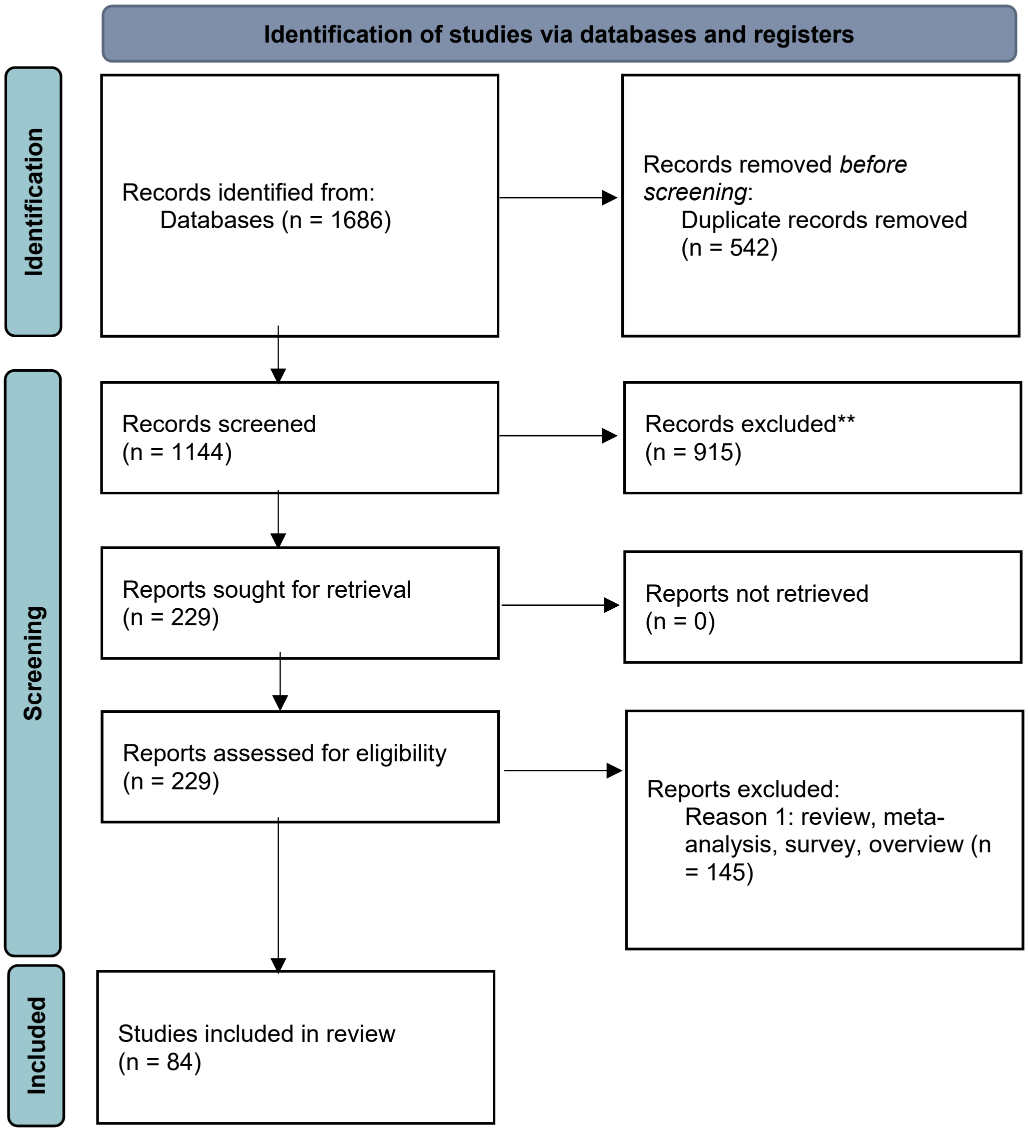

Supplement: Supplementary file 1 [file ijerph-20-04605-s001.zip › Figure S2.pdf]
