# Supplementary figures and images for: From Data to Diagnosis: How Machine Learning Is Changing Heart Health Monitoring
Source: Int J Environ Res Public Health. 2023 Mar 5;20(5):4605. doi: 10.3390/ijerph20054605 (PMC10002005; doi:10.3390/ijerph20054605)

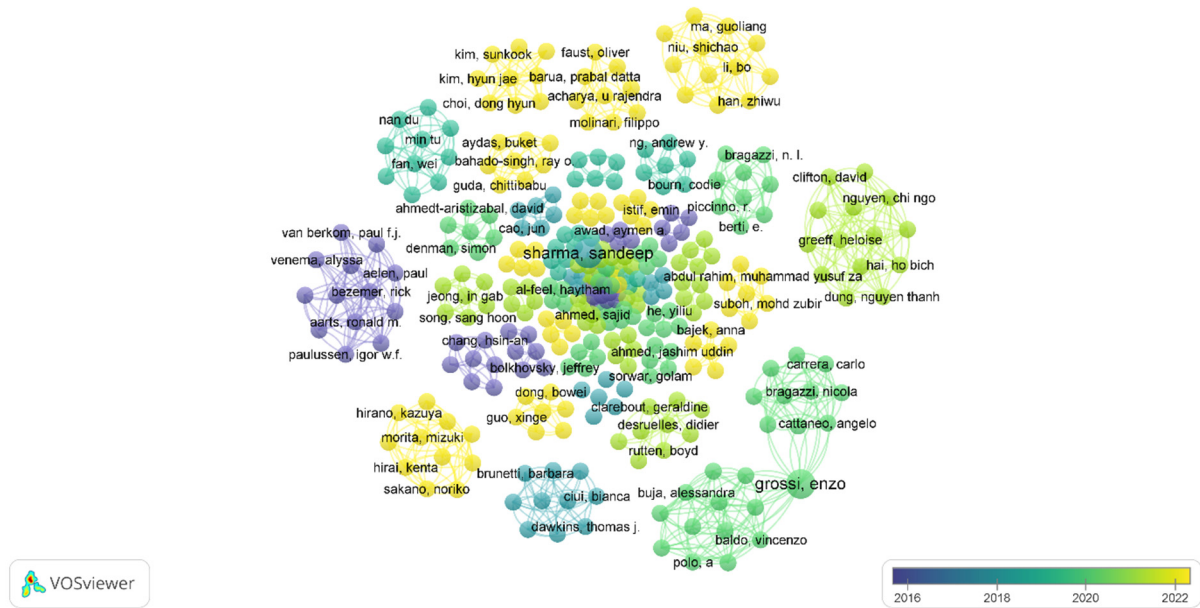

Cluster analysis of semantic web using VOSviewer software

Supplement: Supplementary file 1 [file ijerph-20-04605-s001.zip › Figure S1.pdf]
